# Patient and provider perspectives on how trust influences maternal vaccine acceptance among pregnant women in Kenya

**DOI:** 10.1186/s12913-019-4537-8

**Published:** 2019-10-24

**Authors:** Stacy W. Nganga, Nancy A. Otieno, Maxwell Adero, Dominic Ouma, Sandra S. Chaves, Jennifer R. Verani, Marc-Alain Widdowson, Andrew Wilson, Irina Bergenfeld, Courtni Andrews, Vincent L. Fenimore, Ines Gonzalez-Casanova, Paula M. Frew, Saad B. Omer, Fauzia A. Malik

**Affiliations:** 1 Hubert Department of Global Health, Emory University Rollins School of Public Health, Atlanta, GA USA; 2 Kenya Medical Research Institute, Center for Global Health Research, Kisumu, Kenya; 3 Division of Global Health Protection, Centers for Disease Control and Prevention, Nairobi, Kenya; 4 UNLV School of Public Health, University of Nevada, Las Vegas, NV USA; 5 Department of Behavioral Science and Health Education, Emory University Rollins School of Public Health, Atlanta, GA USA; 6 Department of Medicine, Division of Infectious Diseases, Emory University School of Medicine, Atlanta, GA USA; 7 UNLV Population Health & Health Equity Initiative, University of Nevada, Las Vegas, NV USA; 8 Department of Epidemiology, Emory University Rollins School of Public Health, Atlanta, GA USA; 9 Division of Pediatrics, Atlanta, Department of Medicine, Emory University School of Medicine, Atlanta, GA USA

**Keywords:** Maternal immunization, Health care providers, Pregnant women, Kenya, Attitudes, Developing countries

## Abstract

**Background:**

Pregnant women and newborns are at high risk for infectious diseases. Altered immunity status during pregnancy and challenges fully vaccinating newborns contribute to this medical reality. Maternal immunization is a strategy to protect pregnant women and their newborns. This study aimed to find out how patient-provider relationships affect maternal vaccine uptake, particularly in the context of a lower middle- income country where limited research in this area exists.

**Methods:**

We conducted semi-structured, in-depth narrative interviews of both providers and pregnant women from four sites in Kenya: Siaya, Nairobi, Mombasa, and Marsabit. Interviews were conducted in either English or one of the local regional languages.

**Results:**

We found that patient trust in health care providers (HCPs) is integral to vaccine acceptance among pregnant women in Kenya. The HCP-patient relationship is a fiduciary one, whereby the patients’ trusts is primarily rooted in the provider’s social position as a person who is highly educated in matters of health. Furthermore, patient health education and provider attitudes are crucial for reinstating and fostering that trust, especially in cases where trust was impeded by rumors, community myths and misperceptions, and religious and cultural factors.

**Conclusion:**

Patient trust in providers is a strong facilitator contributing to vaccine acceptance among pregnant women in Kenya. To maintain and increase immunization trust, providers have a critical role in cultivating a positive environment that allows for favorable interactions and patient health education. This includes educating providers on maternal immunizations and enhancing knowledge of effective risk communication tactics in clinical encounters.

## Background

Pregnant women and infants are highly susceptible to adverse outcomes stemming from infectious diseases. The fetal immune system does not fully develop until well after birth; meanwhile pregnancy leads to physiological and immunological changes that alter the mother’s immune system and lessen her ability to effectively respond to infections [1, 2]. According to WHO recommendations and based on the available evidence of safety and effectiveness, most vaccines (including against influenza, diphtheria, tetanus, and pertussis) are not administered until the child is at least 6 weeks old (with the exception of Hepatitis B, Polio and BCG); furthermore, vaccination schedules are not complete until the child is between 14 weeks and 6 months [3]. This presents an immunity gap that can be addressed through the use of maternal vaccines which protect both the mother during pregnancy and the child during the prenatal and postnatal periods [1, 2]. However, low maternal vaccination coverage, particularly in low- and middle- income countries in Africa and Asia where often tetanus is the only recommended vaccination during pregnancy, continues to pose a threat to mothers and infants [4, 5].

There are promising and existing vaccines that have the potential to reduce neonatal and infant mortality rates, however, as mentioned before, the monovalent maternal tetanus vaccination is the only vaccine offered in most developing countries [6]. Despite WHO’s Scientific Advisory Group of Experts (SAGE) recommendations emphasizing maternal influenza vaccination and supporting the continuation and expansion of immunization for pregnant women [7], many low- and middle- income countries have not implemented the recommendation - partially due to concerns about low demand and acceptance coupled with the issue of cost, availability and vaccine approval for inclusion into countries’ schedule [8].

Although some studies have largely attributed mother’s hesitancy to concerns about maternal and child safety that are often based off of misinformation, and also rooted on religious beliefs and cultural norms [9], there is limited information on the determinants of maternal vaccine acceptance in the context of low- and middle- income countries. Given the benefits of maternal immunization that have been observed with vaccines currently in use and the number of new vaccines currently being developed that have the potential to significantly contribute to address the high burden of disease in women and infants [10–13], (for example, a promising maternal vaccine to prevent respiratory syncytial virus (RSV) disease among infants is in advanced clinical trials [14, 15]), it is essential to achieve a better understanding of factors influencing demand and acceptance of current maternal vaccines. This will also facilitate a successful introduction of new vaccines as well as improved uptake of existing vaccines.

Thus, the objective of this study was to describe maternal trust within the patient - provider relationship in Kenya and how it shapes maternal immunization acceptance. Few studies have specifically looked at the role of providers in maternal vaccine acceptance, especially in the context of low- and middle- income countries. For this analysis, we used qualitative research methods to assess the role of patient trust in vaccine acceptance from the perspective of both pregnant women and health care providers in Kenya as an example of a LMIC.

## Methods

This research was part of a larger in-depth, mixed-methods, multi-tiered, national study on maternal vaccine acceptance in Kenya. A research question within this larger study was the role of healthcare providers on maternal vaccine acceptance. This paper focuses on the findings garnered from pregnant women and healthcare providers’ (HCPs) interviews; it specifically addresses how patient trust within the provider-patient relationship affects maternal vaccine acceptance among pregnant women.

### Study sites

Several factors made Kenya a fitting study-site for this project, the first being the recent re-emergence of anti-vaccine rhetoric against the tetanus vaccine. Secondly, Kenya has a large birth cohort (over 1.5 million births in 2012) and has a long standing history / partnership with the CDC that’s resulted in a strong, surveillance system. Lastly, there is considerable regional, cultural, religious, and tribal diversity in Kenya, which enabled our team to gather a variety of perspectives from those who work in the maternal/child health field. Thus, four field sites representing both urban and rural settings were selected, allowing for the examination of cultural, economic, and geographical differences within Kenya (Table 1). Clinics were selected based on their geographic location within the study areas and previous partnerships with the Kenya Medical Research Institute (KEMRI) – Centers for Disease Control (CDC) collaboration.

**Table 1:** Site Descriptions

Site	Description
Mombasa	A major tourist area, Mombasa is located in the south east of Kenya and represents a semi urban setting. Accessibility to healthcare facilities differs on proximity to Mombasa town; Health facilities included Coast General Provincial Hospital (a KEMRI/ CDC influenza site). It is the second largest public hospital in Kenya with a bed capacity of 672. There are about 80 healthcare staff members working in child and maternal health. In 2015, the maternal clinic saw 1882 new patients and 4226 returning patients. This hospital is part of the influenza surveillance platforms.
Nairobi	As the capital city of Kenya, Nairobi represents the urban setting. Accessibility to different types of health facilities (private/ public) is higher in Nairobi than anywhere else in the country. Health facilities included Mbagathi District Hospital which is located in Nairobi right next to KEMRI and CDC Kenya. This hospital has a bed capacity of 320. There are about 53 healthcare workers handling maternal and children issues. The average number of pregnant women seen per month at the hospital is 500.
Marsabit	Located in Northern Kenya, it is a hard to reach and sparsely populated area. It is the most unique of all four locations as it is primarily composed of a nomadic community. Accessibility to any health care facilities is poor. Health facilities included Marsabit District Hospital which is located in the north Eastern part of Kenya. The hospital has a capacity of 86 beds. This location allowed the team to access a different population seen at the other hospitals.
Siaya	Located in Western Kenya and close to Kisumu, Siaya represents the rural setting. Accessibility to healthcare facilities differs depending on proximity to Kisumu which is another major city in Kenya. Health facilities included Siaya County Referral Hospital which serves a large number of rural and low social economic patients. The bed capacity is 200. There are 26 health care workers within the maternal clinic that care for and see about 300 to 400 new and returning pregnant women. It is located about 72 Km from The Centre for Global Health Research at KEMRI Kisumu Field Station

### Participants

Both pregnant women and healthcare providers were selected through convenience sampling methods at clinical facilities (Table 2). Pregnant women were approached and consented at the clinics by study personnel. Healthcare workers were initially approached at the clinic; however, due to a nurses’ strike in 2017 when the data collection was ongoing, the protocol was adapted and most of the interviews were conducted at the homes of the healthcare providers.

**Table 2:** Interview Protocol

Pregnant women: pregnant women waiting for their scheduled antenatal care visits at the clinics were approached by research members and asked if they willing to participate in the study. If they were willing, they were taken to a private room/office designated by the hospital for confidential consenting and interviewing. After allowing time for consent review and answering questions, the study team recorded each interview. • Inclusion criteria: Women aged 15–40 · Women in any trimester; Patient at health facility included in the study; Be willing to converse with others in a focus group format (only for message testing phase); Able to provide informed consent (If participant is illiterate, procedures to ensure full understanding of the research and consent process will be implemented according to international and federal guidelines). • Exclusion criteria: have previously participated in this study; Those who do not or cannot provide consent; Failure to meet other inclusion criteria. • Interview Topics: Interview guide for women included discussions on the following topics: (a) have they had a checkup in the last year; (b) have they received any vaccines that they can recall; (c) if they have received vaccines, they will be asked about their understanding of the vaccines they received [e.g., do they know what the vaccine prevents against, did they get the vaccine just because their parent/doctor told them to]; (d) comfort discussing sensitive topics with their doctor and parents; (e) awareness of maternal vaccines; (f) information from peers about maternal vaccines [friends’ vaccination status, anecdotal side effects, discussions on social media, reasons to get it/not get it]; (g) discussions with parents/guardians about maternal vaccines; and (h) motivating factors to be vaccinated.	
HCPs: providers working at the antenatal care were contacted ahead of time to arrange interviews for times that would work best for them. During this phase of data collection, HCP were on a nationwide strike. To mitigate the effects of delayed data collection, study team members organized interviews outside of clinic. • Inclusion Criteria: Currently working at the selected study sites; Current physician, nurse, nurse midwife, community health worker; Able to provide informed consent. • Exclusion Criteria: Those who do not or cannot provide consent; Failure to meet other inclusion criteria. • Interview Topics: The semi-structured interview guide for providers included discussions on the following topics: (a) proportion of patients they estimate have received or refused maternal vaccines; (b) times at which they recommend maternal vaccine; (c) practices regarding immunization history verification (e.g. immunization information system); (d) barriers or reasons for refusal cited by parents/patients; (e) perceived ability and methods used to address these barriers/refusals; (f) comfort discussing vaccine recommendations with their patients; (g) existing efforts of reminder/recall for maternal vaccinations; and (h) knowledge of Tdap vaccine effectiveness and safety.	
Participant observation and Facility Profiles: Non-structured observation of pregnant women and HCPs were also conducted within the clinic. The research staff took detailed field notes to examine patient-administration, patient-patient, patient-provider relationships, dynamics of provider-government officials, and provider-provider, provider-patient, and provider-administration interactions within each of the selected sites. Interview notes and observations notes were used to edit the guide as needed. Notes about each facility, e.g. patient flow, vaccine storage and supply chain, etc. were also typed up over the course of interviews.	

### Study protocol

Semi- structured, in-depth interview guides were developed using grounded theory [16] by the study’s lead anthropologist with input from the study team, in-country partners, and a scientific advisory committee. To understand the social determinants of maternal immunization acceptance among pregnant women in Kenya, open-ended questions explored socio-cultural practices customs, values and beliefs (Additional files 1 and 2).

During the initial pilot-testing phase, the guides were reviewed, revised, and updated as new themes emerged. An expert anthropologist trained in-country research team members in qualitative methods including protocol adherence, screening, consenting, qualitative interview methods, transcription and translation. The inclusion and exclusion criteria are described in Table 2. Interviews were conducted in either English, Swahili, Kikuyu, Luo, or Borana depending on site and preference of the interviewee. Interviews lasted approximately 30 to 60 min, depending on the extent of the discussion, and were audio recorded. Interviews were conducted in teams of two, allowing for an interviewer and note taker (Table 2). Both KEMRI IRB and Emory IRB approved the study protocol. CDC IRB reliance on Emory’s IRB was obtained.

### Analytic approach

Interviews were transcribed and translated (when necessary). The analysis was done using N-Vivo 11.0 qualitative data analysis software. Identifiable information such as names, dates, and addresses were removed from both the recordings to maintain participant confidentiality.

Prior to coding, the qualitative research team developed two codebooks including one for providers and one for pregnant women’s interviews. Once the codebooks were completed, codes were applied to the transcribed interviews; major thematic content emerged from this process. Intercoder reliability was performed among three coders participating in the coding and analysis. A kappa coefficient of ≥0.80 was considered a minimal cut-point for high intercoder agreement among coded content to maintain rigorous qualitative research standards. After 4 rounds of intercoder testing, the team achieved k ≥ 0.80 agreement on codes used in this analysis.

## Results

A total of 328 pregnant women and 112 HCPs, including nurses and clinical officers, were interviewed. Of the 112 HCPs, 42 HCPs worked in only public facilities while the rest worked also at private facilities. Our primary themes were patient trust, patient health education and provider attitudes towards patients. Additional quotes corresponding to the themes and subthemes are depicted in Tables 1, 2, 3, 4, 5 and 6.

**Table 3:** Patient trust and views on patient autonomy – HCP Perspective

Subtheme	Quote
Expressed Trust	*“When they come, they just accept what the doctor gives them because they believe the doctor is always right. They have never challenged us by asking ‘why are you giving me this vaccine and not the other one?’”* *“May be sometimes they do not have that chance to say no because they look at me as their savior, the last person for them and everything I tell them they do believe is right”* *“Actually, patients just come for ANC clinics and it is us, the health care providers who decide what is deemed fit for them; they do not ask for anything.”*
Respect for autonomy	*“We believe that the client is always right. Therefore, after taking our time and proving a detailed health talk and a woman still refuses to be vaccinated, we do not force them. Clients have the right to accept of refuse medication. We honor their requests and what they believe in. we always give them a lot of information anyway.”* *“Most of the mothers who come here for the vaccine know that they have to be injected. Others had not received the tetanus vaccines in their previous pregnancies so they do not see the importance of the vaccine. We explain to them the importance and tell them to go think about it and come back because we cannot force them.”* *“Normally after you have done all the necessary services you will tell the mother now it is time to give you the vaccine we normally tell the mother that* “*I want to give you a tetanus injection” during that time she has the right to tell you if she does not want the injection or she just accepts. However, we have never had a case in which a mother declines.”*
Authoritative approach	*“We tell them what they need to have so I do not think decision-making is on their side so they just receive it.”* *“I: Okay, once you make available, vaccines in your facility especially the tetanus vaccines, do you think mothers get ample time to make a timely decision whether or not to receive the vaccines in your facility?* *P: They have no choice, we just tell them it is mandatory and it is good for them.”*
Sources of trust	
1. Education	*“Yes, they normally have time to accept because by the time they leave their homes to come to the clinic, they are very sure the doctors or the nurses or health care providers know more than they do, so they will just do what the health care ask them to do”*
2. Altruism	*“I think it is the norm of the clients where you find that patients always feel that the doctor is the one who knows what she should receive as they believe the doctor will do the right thing.”*

**Table 4:** Provider Attitudes towards patients (approachability)

Subtheme	Quote
HCP Perspective	
Impact of attitudes towards patients	*“The other reason* [barrier] *could be maybe the way you talk to them when they come for their clinic here. For instance, if you talk to them rudely, they may not come back her.”* *“I think that is attributed the service we provide. We talk to them politely. Even if the client is unhappy with you, you must find a way of working towards that. In my facility, we sometimes provide tea, snacks and water for clients.”* *“The nurse kept accosting the patient about the time of coming for ANC clinic and her age. A lot of words were told to this the teenage mum that made me doubt if she would dare come back for the services though eventually got the services and ANC book provided.”* *“For one is the attitude of the caregiver, the availability of the vaccines and the availability of time and staff. If we are many we will shorten the waiting time. And my attitude also if I have a poor attitude, I will discourage them but if it is good, they will encourage others to come”*
Evolution of patient-provider relationship	*“Uptake depends on the attitude of the healthcare provider. Most mothers have issues at home and how you handle them matters a lot. When a mother walks into your clinic, they are walking to someone they believe in their heart will help them. The information you give this mother may change or break her. I believe it is all about the attitude and approach towards these clients and the education you give them.”* *“These cultural practices are still there but there was a lot of force from the administration chiefs ensuring they are given by force, which should not be the case. The community should be taught then consent after understanding and receive the vaccines.”* *“Some of them [providers] would be hindrances because they would not engage mothers when they are making decisions concerning vaccines dates. Some will put vaccine date to their convenience without considering the mother’s side. Those are open hindrances. There are some people who say on giving vaccines on specific working days without taking care of mothers who work from Mondays to Fridays.”*
Patient’s desire for information	*“They will say it is new, we want to know its constituents.”* *“They normally ask the importance of those vaccines, how the vaccines help them, if there is any adverse effects and what would be done in such a situation.”* *“They ask on why we give the vaccines and what they prevent against. Therefore, whenever we give vaccine, we first seek their consent, give the reason for administering and talk about their importance.”*
Effect of Time -constraints on education	*“For now, I can say they do not get enough time since I am alone, overwhelmed and take shortest time possible with them. They do not get enough time.”* *“So I am the type of person who has to give the mother the information she needs. So I cannot know whether my colleague in the other room is giving that information. Sometimes I cannot blame them because you find that three benches are already full and all the women are waiting for that one person to attend them. You will find that things there are not going as intended. The education is usually not there as such. But if we had enough time... so even if there is no ample time you have to give information of the very important things which the mother has to take home from that room.”* *“I think that is the only challenge because for vaccines we have everything we need like syringes and the like. If we can get additional staff, then it would help us a lot because I may take shortest time with clients since people waiting at the queue are very many thus making me rush and where I would have explained more about pregnancy may not be possible.”* *“I think the most important is the human resources because if you are only one person, you become overwhelmed. Most of our clinics are usually overwhelmed especially on Thursdays and Mondays. This happens especially in the child welfare clinic where you have to take care of immunization for children and at the same time pregnant mothers. It is hectic. That is where quality is compromised because you hurry to clear the queue.”* *“So you find that the nurse has a lot of work because she has to be here, she has to be in the OPD (Outpatient department) and also coordinate all the activities in the hospital. And this being a sub-county facility, she has to attend to all the visitors who come because we have the sub county officers. So the nurse will not find enough time to attend to the clients so it makes the clients to wait for long. We have to finish the other side and come to help this sides. So if you are in a position to add us one or two or more it will be good because this is a health center and it cannot be managed by two people.”*
Pregnant women perspective	
Importance of provider attitudes on trust as expressed by facility choice	*“I focus on the reputation of the hospital and the way the doctors treat people.”* *“I look at how patients are being treated. You will come to a certain hospital after you have heard people praising it.”* *“P* *: The staff here are very kind and attend to us well. Their services are also good.* *I* *: What about [name] Hospital?* *P* *: Sometimes the nurses are very harsh which makes us feel uncomfortable”* *“You know there are places that you can go to and you are attended to in a hurry and maybe you have a particular problem. Instead of somebody listening to you, he/she starts despising you.”* *“* *I* *: What is it that will motivate you to go for vaccine?* *P* *: Pregnant mothers are sometimes turned away from hospitals when they go to deliver if they never attended the antenatal clinic.”*
Accessibility	*“Well actually the distance from our home to this place is quite long, but I have no choice but to come here. Clinic services for pregnant women are available only in a large facility like this and not in small health centers and dispensaries like the one in our location.”* *I:“In case you become sick, and you want to go to the hospital, what can you put into consideration?* *P* *: When I become sick and I want to go to the hospital?* *I* *: Yes.* *P* *: I must have means of transport to the hospital. (Laughs).”* *“However, this side you must be sick is when you come or when your day for clinic reaches is when you come, a times you are sick and the place is far you will just be force to persevere.”* *“I just go to the hospital that is close to me.”* *“Yes, this general hospital is closer to me than the others are.”* *“I* *: Okay, and what do you do if you get sick?* *P* *: I go to the hospital* *I* *: Which hospital?* *P* *: Any that is close to me”* *“You go to the nearest hospital when you are sick.”* *“* *I* *: why this one? What attracts you here?* *P* *: It is near where I stay”*

**Table 5:** Patient Health Education

Subtheme	Quote
HCP Perspective	
Effects of myths and misconceptions (“Rumor mill’)	*“We recently had a challenge with polio and other vaccines that were being said to bring infertility. Those who do not get a chance to talk to a healthcare professional to enlighten them about these myths end up believing what they are told out there. The public sector has these challenges.”* *“For example, sometimes back, there was a serious debate between the ministry of health and Catholic church. The church was against the tetanus toxoid. The catholic church argued that the vaccine was meant to sterilize female populations. The issue was all over the internet and social applications. I think vaccination efforts did not reach their targets. There are some who also complicated the issue justly to scare more people away from vaccination.”*
Education as a tool for demand creation and reinforcing/ building trust	*“When communicating to them we need to tell them about the importance of vaccines and insist for them to receive. If you do not tell them about the importance, then they will not take. As you know that the patients normally believe in doctors and they will do whatever the health care provider suggest to them to do.”* *“We should have somebody at the triage, one in the child welfare clinic, one in the ANC clinic, one in the family planning clinic and one in the PMTCT. This would help us give mothers time to ask questions and also give as time to address them. This will also make the mothers to be comfortable with us since I will not be rushing through but will have adequate time to give the mother’s health information. Pregnant mothers need a lot of information and especially first time mothers who could be having wrong or outdated information”* “*There are pamphlets with pictorials about the effects of tetanus infection. When we show women such pictures, they understand the importance of tetanus vaccine and accept vaccination. You realize that TT uptake is increased. They are confident with what we tell them and we are also confident that their attitude is positive. This is evident in the fact that they come in numbers for the vaccines. In some cases, they come from other hospitals. They trust us.”* *“The first thing, which I appreciate about health information given to the mothers; is that at least they know that there is an antigen which they should be given and they appreciate about that antigen. Secondly, they know the importance of attending the ANC clinic because if you ask them why they always come to the clinic, they will tell you that “I come because I need to be given tetanus” so basically, the message you give them has a positive impact.”* *“Barriers. Mostly because most of the issues that normally come up are always myths, we try to debunk them by trying to tell them the facts. Like somebody believing that when they are pregnant they can’t get injected, you talk to them, you tell them the importance and we also expose them to know the side effects though in most cases the side effects are always very minor and I have never met an adverse reaction with the vaccines. So we always try to talk to them. We let them know the facts so that they make an informed decision. Some come when they have bad opinion about vaccines but they end up getting it, having been given the facts.”* *“Basically, it is the health information. We give them a group health talk outside then when they come in, we have one on one health talk. However short it is we make sure we tell them the importance. By the way, I have realized they know the importance of tetanus. Once we give them the information on the importance of the vaccine so we do not expect refusals.”*
Community buy-in	*“More publicity. These can be done by women who have received vaccination telling fellow women, pregnant and non-pregnant alike about the importance of vaccines. Government officials like chiefs and village elders can also play their part by organizing barazas-(Gatherings/meetings organized by the local chiefs to address issues) for all women where they will be educated on vaccines.”* *“They should also have the information because one mother will tell another and that is how information flows.”* *“The moment they know what we are doing, they become our ambassadors in most cases. They take that message home. When you do something right to one patient, you will help like five of them because when she goes out there, most of them share their experiences.”*
Pregnant women perspective	
Effects of myths and misconceptions	*“I:* *Why do other people refuse vaccines?* *P:* *Others just take it lightly, others because of religion and others think it is wasting time.”* *“I:* *Have you ever refused vaccine for yourself before?* *P:* *Yes. There was one that brought lots disagreements. You know I am a Catholic … It was also in church but when we saw our leaders arguing with the government saying the vaccine is not good that it has other things. I rejected that one. By that time if you went to the clinic you could be asked to be given the vaccine but I refused.*
Importance of education on vaccine acceptance	*“Yes, the information on children’s vaccines was helpful. When the healthcare providers came administering the measles vaccine, a measles outbreak had just occurred. The healthcare providers informed us that those who will get the vaccine before contracting measles will be safe. And because of this information, even those who had never been vaccinated before came for vaccination.”* *“Why I received the tetanus vaccine was, it was well publicized, the information that came with it was okay, those people who were also giving it out, I believe they were professionals because they also had tags. Before they give you the vaccine, they had to explain what would also be compared with the information we had before; to me that was okay, I did not even need a second thought about it, yes.”* *“Mothers should be educated on vaccines first and then they can choose. As I told you earlier, some mothers refuse vaccines because of some misconceptions they hear about vaccines. There was a time everyone thought that polio vaccines would kill a child. I do not think the government can kill all the children in Kenya, I believe there have a conscious too.”* *“They said that children will be prevented from serious physical handicaps and polio. Since I do not have more knowledge than doctors, I accept to have my kids vaccinated.”* *“I* *: Let us say she refuses and the child happens to be infected by the disease that would have been prevented and remember the child has no capacity to take her/himself to the facility for the services, so what do you suggest to be done to such a parent?* *P* *: Just give her good pieces of advice.”*
Desire for health education	*“I come here because of the services they provide but mostly I like the guidance and counseling they provide.”* *“AMREF together with the nurses usually go round the villages to vaccinate the children so I ask them about it that is where I learnt from. I have to ask because I will find myself in that situation where my child has to be vaccinated so I need to know.”* *“I think you should put more advertisements on radio and television. There should also be caregivers to teach us when we come here in the morning. But you find that when you come to the clinic, you might sit at the reception for even an hour without anybody attending to you. When they finally attend to you, you just go home. Ever since I started coming here, we have only been educated once. Maybe it is me who comes early and they do it later.”* *“For example, I went to a private hospital but I was not given any vaccine or advice as a pregnant woman. I was also not asked any question as a pregnant woman. They only tested me and filled the form and by that time I was in great pain. That is all they did. So I thought that if I come to Mbagathi Hospitals, I will get vaccinated and get advised. Like today I have been advised to start preparing for the delivery of my baby. I have been told to have a razor blade, string and money. I have received advised which I would not have received in private hospital. I have also been told that I need to eat well as a pregnant mother and to also use folic tablets for me to have enough blood in the body. I would not have received such advice in a private clinic.”* *“* *I* *: Between government hospitals and private hospitals, where would you prefer to be vaccinated?* *P* *: Government hospitals because they educate a lot on vaccines.”*
Fear of reproach	*“There are some doctors that when you ask them questions, they will also ask you, if you came to be treated or for questions; it becomes difficult to interact with such ones.”*
Time constraints	*“I do not ask because I find many women on the queue. You do not have the time to ask why do you so you just agree to be injected so that you go home.”*

**Table 6:** Expressed patient trust - Pregnant women perspective

Subtheme	Quote
Explicit trust	*“I* *: Why would you trust the doctor?* *P* *: If there could be no doctors I could not be even alive. They have really helped me.”* *“Since I am sick, I will trust no one but the doctor, he will screen me and then tell me what the problem is.”* *“You see you can discuss with people from home but they are not doctors, they will listen to your issues and at long last refer you to go and see the doctor because they have no knowledge on the same. At the hospital, the doctor will examine, test and know the cause of the problem while at home people will tell you it is just malaria; I think that seeing the doctor is the best thing.”*
Direct impact of trust on acceptance	*“I will comfortably receive vaccines here at the hospital because it has the right personnel. I will not take it anywhere else where there are no experts.”* *“I believe that anyone who gives me vaccines knows why he is doing that; I believe he/she has gone to school and understands this issue better than I, and I have no reason to refuse as long as the vaccine does not kill me, and as long as my health improves.”* *“* *I* *: why wouldn’t you refuse to receive vaccines?* *P* *: This is because it is important to our body and especially if it’s given by healthcare providers who are experts and informed then I cannot refuse.”* *“I will accept because it is recommended by the doctor since that is their profession hence they have knowledge as to why they bring that new vaccine.”* *“Some people even think if they are vaccinated they would come impotent like in men even women would not give birth like the case of tetanus, what other women used to say is that women have born a lot of children in Kenya, so that vaccine is a way of birth control that was going around which I also heard but I said if it is so that is what the government is planning which I don’t think is true, so me I just went ahead and received. It has not stopped me from conceiving, yes.”*
Reasons for trust 1. Respect for provider’s education 2. Government authority 3. Belief in provider’s altruistic motives	*“I trust them because these are people who have knowledge in that line, it is something they have studied, yes, they have been tested on that so they stand to convince me that I can rely on them.”* *“I will accept because it is recommended by the doctor since that is their profession hence they have knowledge as to why they bring that new vaccine.”* *“I will rank the hospital as the first, because the information you get from the doctor has no doubt since the doctor has the knowledge.”* *“I will still admit because it is a government command”* *“Yes, I do believe that vaccines are safe because the government cannot bring something that will harm us.”* *“So I knew it was something that was initiated by the government so I did not see the need to debate it that is why I went ahead and took it, yes.”* *“Because I know that when the Community Health Workers comes they come with the doctors and secondly when I’m in the hospital I trust all of them because I have never seen a doctor without a tag and that will prove that what they are doing is what has is authorized by the government.” “However, you cannot refuse yet it has been rolled out by the government and doctors. A doctor cannot prescribe harmful drugs, unless a quack.”* *“I know the doctor is the one who treats people so if he gives me the vaccine I know it’s a correct thing, because the doctor cannot wish to harm anyone.”* *“I* *: You have said you trust the doctor why do you trust the doctor?* *P* *: Because they have already devoted to help people.”*
Preference for public hospitals	*P:* *I go to government hospitals.* *I:* *Why?* *P:* *Even if you do not get medicine, the medicine they prescribe is good because they are not looking for money unlike private centers where they tell you medicine are original and you are left wondering whether there are fake and original medicine. I worry about that.* *P* *:You know everybody has their own decision but for me I decided all my medical advice and services I will be getting from a public hospital and that is why I am here.* *I* *: So what is good with this public hospital that is not in the private hospitals?* *P* *: In public hospitals you are sure, somebody will not cook. You are sure of services and it is not business so if you have a particular problem you will be told that you have this particular problem, there is no exaggeration or undermining that is why I choose public hospitals.*

### Health care providers’ perspective

1. Patient trust in providers and resulting ethos

Multiple providers reported that their patients would accept whatever they recommended because their patients completely trusted them (Table 3). Providers believed that this trust was primarily rooted in the providers’ social position as a learned professional holding a great deal of knowledge about health. Additionally, providers believed that patients trusted that providers would always do what was right for them (Table 3). This perception of trust manifested in two different clinical approaches: providers who were presumptive and administered the vaccine to their patient without communicating what it was; and those who thought it still important to inform their patients what vaccine they were receiving prior to administration.

*“They have no choice, we just tell them it is mandatory and it is good for them.”*

**VS**.

*“I think, when women come to the health care provider they have trust in them. Since they trust us, they will also trust what we tell them. It is now our work as the health care providers to give convenient information to these women so that they can go back to their homes satisfied.”*

In both examples, providers explicitly or implicitly expressed perceived trust from their patients. However, the first provider had a more paternalistic view while the latter expressed the need to inform their patients. Ultimately, regardless of their personal belief systems, all providers recognized the amount of power this trust bestowed upon them and believed patients would not typically refuse to adhere to their recommendations (Table 3).

2. Provider attitudes towards patients (respect and approachability)

Although patient trust was implicit and very few providers reported having ever had patients refuse to be vaccinated, providers generally acknowledged the importance of their own personal attitudes for patient trust and vaccine acceptance when addressing pregnant women (Table 4). While some providers still practiced authoritative approaches, particularly in the rural areas, others heeded that the expectation of patient deference was no longer universal.

*Nowadays, people do not harass mothers like in the previous years … In the past, people used to be blasted by the nurses or whoever was giving the services whenever they asked questions. Those days are long gone. It is always good to ask why you are being injected* *.*

Some providers noted a shift in the evolution of the patient-provider relationship to one that needed open communication and respect for continued trust and acceptance of vaccines (Table 4). Many providers said that patients would prefer to be consulted and informed prior to receiving the vaccines (Table 4). However, even while acknowledging the need for positive attitudes and educating their patients on what they were receiving, many reported not being able to always brief their patients in practice; largely due to heavy workloads and time constraints.

“*Maybe when a health care provider is in a hurry or is being overworked. You may find a long queue at the ANC waiting for vaccination. The nurse there may not have time to discuss much with every client about the vaccines. Sometimes they issue orders for the mothers to queue and get vaccinated. These are situation which may happen when there are several mothers at the clinic. This can cripple vaccine uptake since there is no time for explanations.”*

3. Patient health education

Providers reported various religious and cultural barriers to vaccine uptake. However, providers highlighted the negative impact of recent controversial remarks by religious and political groups about the tetanus vaccine. They claimed that the vaccine led to infertility. These allegations resulted in increased vaccine hesitancy (Table 5). When asked how to mitigate these effects, providers touted the importance of health education as a way through which they could dispel these rumors and increase acceptance.

*“But with continuous education given, the posters, you find that the number that come to access vaccination is high. For example, if you forget to give they will ask you for the vaccine.”*

Although anti-vaccine rumors reduced patient trust in both providers and vaccines, provider responses suggested that there was still enough trust left among pregnant women to allow for the use of health education as a reinforcement tool.

*“They always appreciate as long as the information that is being given to them is from somebody from a medical profession and whom [they] trust.”*

According to the providers, educating the patients about the importance of vaccines would not only increase vaccine acceptance but it would also reinforce patient trust in providers and reinstate it in situations where it had dissipated. Additionally, providers reported that health education reduced default for subsequent vaccination and could also promote communal buy-in in cases where these women became vaccine advocates within their community (Table 5).

### Pregnant Women’s perspective

#### 1. Expressed patient trust

Pregnant women supported HCP views on trust. They explicitly said that they would accept whatever is recommended by HCPs, even if they didn’t know what it was. There were multiple women who reported not knowing what was administered to them but accepting it anyways because it came from a “doctor.”

*It is us who need it, and we don’t know why we need it, so there is no way we can refuse. In addition, you cannot dispute what a doctor tells you, especially on something that he has taken years to train for. Even if they inject you with poisons or any other substance other than the vaccine, you wouldn’t know and you won’t have any say.”*

Pregnant women describe a broad range of providers including healthcare volunteers, doctors, nurses, chemists, and outreach healthcare workers as “doctors”. Pregnant women did not always differentiate one from the other but when they did, they would often use the hospital as an identifying marker between providers. They would either describe the provider as the doctor walking around the neighborhood (community health workers), or as the doctor in the hospital (physicians, nurses, chemists). For the pregnant women who made a distinction between the different types of providers, trust was sometimes expressed more towards those providers who worked at the hospital.

*“I believe it is safe if it is from health centers but not out there because you may not know who sent them and their motives. I would rather come to the hospital to confirm if there is a vaccine being given”* *.*

When asked why they trusted HCPs, most pregnant women replied that they trusted providers because 1) providers are learned about health and are the only ones who can decipher their illnesses and treat them accordingly, 2) providers have institutional authority from the government to guard their health and 3) providers are healers and caregivers with honest motives (Table 6). Most notably, in some of the cases where patients showed hesitancy towards vaccines, patient trust in the government, superseded that mistrust (Table 6).

#### 2. Provider attitudes towards patients (respect and approachability)

Once again, though trust in providers was consistently expressed, pregnant women also spoke about the importance of provider attitudes on facilitating trust and vaccine acceptance. Mirroring HCP views on provider attitudes, pregnant women shared that attitudes greatly contributed to where and when they would choose to go seek medical care.

*“Personally I would not have come back here if it were not for my condition because of my first experience here. Because when you come to the clinic, you expect to find friendly people who are ready to help you. But if you come and find somebody who is arrogant, one who has I do not care attitude and it is like you are bothering them, I will prefer to go somewhere else where I will find somebody who will understand my condition.”*

It is however important to note that provider attitudes were mostly considered as a factor in vaccine decisions by participants living in urban areas where there is an abundance of facilities available. Most participants living in rural areas often reported having little choice in where to go and considered distance and cost much more than they did provider attitudes (Table 4).

#### 3. Patient health education

Women also noted the importance of health education in their decisions to accept the vaccines. While there were many who did not know what was being administered and did not care about knowing more about the vaccine, many others reported that having more information about what was being administered increased trust in their provider as well as the vaccine.

*“I come here because of the services they provide but mostly I like the guidance and counseling they provide.”*

On the other hand, there were many pregnant women who wanted more information but stated that they were unable to receive it. When asked why they didn’t inquire about what they were receiving, patients report either being scared of being admonished for speaking up / asking questions, or not having an opportunity to ask questions due to time constraints (Table 5). The lack of information and hostile provider attitudes may have not always hindered vaccine acceptance for most women, but some did say that it lessened their trust in HCPs:

*“Even if it were you, you would be scared. We believe that if you are a know-it-all, they may even harm you. It is like telling the doctor ‘you did this and it is not done like that.’ You are sure that is not how it is done but because he/she wants to show you that he/she is there for that job and knows more than you do people say that he/she can harm you because you do not know what you are being injected with.”*

## Discussion

In this study, we assessed how pregnant women and antenatal care providers perceive patient trust in their relationship and how, from their perspectives, it affects maternal vaccination in diverse areas from Kenya. The central result from this study was that both pregnant women and providers recognize that high trust is placed on the health care providers to make decisions about maternal immunization. A concern that was identified by both sides was that often, this trust in combination with time constraints leads to the use of ‘authoritative’ approaches from the providers’ side who sometimes vaccinate without providing information to the women. While this could be compared to the presumptive approach that is recommended in the US and other high-income countries, failing to provide pregnant women enough information to make informed healthcare decisions could lead to a deterioration of this trust. In this sense, women reported not feeling empowered to request information, but still trusting the providers to make vaccination decisions. In turn, some providers acknowledged recent changes where women are now allowed to request information and stressed the importance of shifting towards a relation that allows better communication and respect for the patient. This could also help address other sources of misinformation that can increase maternal vaccine hesitation.

A common theme among both pregnant women and providers was the concern that increasing misconceptions disseminated by some religious and political leaders [17, 18] could have led to increased vaccine hesitancy and mistrust in Kenya. Some of this misinformation included warnings against vaccines being used as means for sterilizations. While it is hard to determine direct causality, these statements occurred in parallel with a 16% decrease in women who had booster doses between 2013 (77%) and 2016 (61%) based on data from the Kenya Demographic Health Surveys [19]. In this context, our results showing the strong trust of pregnant women on their providers to make decisions about vaccination highlight an opportunity to preserve and leverage this relationship to improve to improve maternal vaccine acceptance for existing and new maternal vaccines (e.g. RSV).

We identified two main factors that could be utilized to improve this relationship through increasing trust: patient health education and improved patient- provider interactions. The association between patient trust, provider attitudes towards patients, and patient health education is cyclic. Both pregnant women and providers expressed that pregnant women wanted to know more about vaccines, and that patient health education can increase trust in both the provider and the vaccine itself. Conversely, pregnant women conveyed that they only trust vaccine information if it is relayed by providers. However, the amount of trust that is inherently present between patients and providers is reportedly being hampered by poor provider attitudes towards patients. Both providers and pregnant women stated that inherent patient trust was rooted in a fiduciary relationship: patients trust that providers know more about health and consequently transfer autonomy to the provider. A fiduciary relationship, as defined by James Marcum, is one where trust stems from the provider’s expert and technical knowledge [20].

Although patients were not opposed to providers making health decisions for them, they voiced their frustrations at how providers sometimes treated them. Many women shared that staff attitudes greatly contributed to if, when, and where they chose to go seek medical care. Similar findings were previously reported in a study that looked at patterns of childhood vaccine acceptance in Malawi, India, Ethiopia and the Philippines [21]. Since maternal vaccination with TT is provided through antenatal care visits, if providers’ attitudes discourage women from attending the visits, decreased maternal vaccine coverage could be one of the many negative consequences of this miscommunication. Our results highlight the importance of working towards respectful antenatal care as central to improving maternal vaccine uptake in Kenya.

Aside from facility choice and impact on access to antenatal care, these attitudes also hampered effective health communication between patients and providers. Pregnant women cited rude and intimidating behavior from providers as factors hindering their willingness to ask questions or come back for subsequent treatment. Providers corroborated this view and attributed this behavior to historically paternalistic approaches and heavy workloads and time constraints.

These results suggest that patient education and provider attitudes towards patients are imperative for the growth of this trust and are interrelated in a cyclic fashion; patient health education reinforces patient trust in providers while providers’ attitudes towards their patients can either reinforce or hamper that trust. We recommend that, in addition to improving their attitudes towards their patients, providers should learn effective risk communication and how to facilitate open communication. This promotes the patient’s knowledge and self-efficacy which, as evidenced by the health belief model, improves health outcomes [22]. Governments can facilitate these changes by including modules on patient health communication during continued medical education (CMEs) for providers. Additionally, given our data’s illustration of the impact of provider time constraints on patient- provider interactions and patient health education, facilities can mitigate these effects by using the community health volunteers to educate pregnant women on vaccines.

In addition to trusting the provider because they consider them highly knowledgeable, pregnant women said they trusted providers because they had governmental authority. Pregnant women believed that providers would not administer anything that was harmful because the government would not harm them. Sometimes this trust extended to the types of health facilities they chose to frequent: pregnant women showed more trust towards public health institutions than private health institutions. This is an important revelation given that low trust in governments is considered to contribute to the global hesitancy of vaccination [23].

One of our limitations is potential selection bias. The pregnant women in our study were recruited during their antenatal care visits at the health facilities. By virtue of them already being at the hospital, they may already have relatively high trust /little resistance to seeking care. Similarly, we only interviewed women seeking treatment in public facilities; those who attend private facilities may hold different views that were not captured by our study. However, most women in Kenya attend public facilities and the large sample size, especially for a qualitative study, could have offset some of this limitations. Additionally, most public health care providers were on strike during this phase of our research which could have also influenced their answers, since they were not practicing and perhaps had a particularly negative outlook. However, we were able to complete the data collection by meeting providers at their convenience, and capture the views of healthcare providers under real world conditions.

## Conclusions

Our study highlights the importance of the patient- provider relationship as a facilitator for maternal vaccine acceptance in Kenya. Maintaining and improving trust within this partnership is extremely important for patient compliance. We argue that health care systems cannot rely on patient deference for treatment compliance, especially in a changing context where trust in the system might decrease over time (as seen in other countries like the United States [24]). Recommendations to foster maternal vaccination acceptance moving forward include motivating providers to allow open communication with pregnant women, and providing information to improve patients’ knowledge and understanding of the importance of vaccination during pregnancy. Important next steps are to provide this information to policymakers and healthcare managers to try to implement some of the recommendations written herein.

## Supplementary information

**Additional file 1.** Interview guide for healthcare providers.

**Additional file 2.** Interview guide for pregnant women.

## Data Availability

This analysis was primarily based on qualitative data. Some data may be available upon request after reviewing for confidentiality. Please contact Dr. Gonzalez Casanova to request the data (igonza2@emory.edu).

## Abbreviations

ANC: Antenatal care providers
CDC: Centers for disease control
CME: Continual medical education
HCP: Healthcare providers
IRB: Institutional review board
KEMRI: Kenya Medical Research Institute
WHO: World Health Organization
