# Supplementary material for: Patient and provider perspectives on how trust influences maternal vaccine acceptance among pregnant women in Kenya
Source: BMC Health Serv Res. 2019 Oct 24;19:747. doi: 10.1186/s12913-019-4537-8 (PMC6813986; doi:10.1186/s12913-019-4537-8)
Supplement: Supplementary file 1 — Additional file 1. Interview guide for healthcare providers. [file 12913_2019_4537_MOESM1_ESM.pdf]

Healthcare Providers

Introductory Questions:

1. What is your job description?

2. What is the highest level of education you have completed?

Current Vaccine Information:

1. Which maternal vaccines do you offer here at the clinic?

   PROBE: Why? Why not? Since when?
   PROBE: What do patients ask for?

2. Do you feel you have enough demand to suggest vaccines to people?

3. What types of resources does your clinic have (or do you think would be needed) to deliver vaccines at your clinic? Both, Educational and Logistical.

   PROBE: In terms of who or what?
   PROBE: Patient-focused educational resources?
   PROBE: Provider-focused resources?
   PROBE: How many resources are needed?
   PROBE: Storage resources?

4. At what times do you start talking with your patients about maternal vaccines?

   PROBE: Gestational age

5. Do you feel you have enough information to confidently discuss vaccines with your pregnant patients?

6. Are there vaccine-related educational resources that you provide to patients?

7. Do you normally provide vaccines or vaccine related information to pregnant women?

8. What do you feel is the impact of these materials on your patients?

   PROBE: What is the patient's reaction?
   PROBE: Do you think these materials work?

9. When you make vaccines available at your practice, for example the tetanus vaccine – do you think women get enough time to review the information and then make a timely decision?

Patient attitudes:

1. What proportion of patients do you estimate have received or refused maternal vaccines?

2. How frequently do pregnant women ask specific questions about vaccines in pregnancy, vaccine preventable diseases, or maternal and childhood vaccines?

   PROBE: Perceived ability and methods used to address these barriers/refusals
   PROBE: Barriers or reasons for refusal cited by patients
   PROBE: Are women asking for new vaccines?

Vaccine knowledge of healthcare providers

1. Do you feel that maternal vaccines are harmful? Why or Why not?

2. What do you know about the Tetanus vaccine?

   PROBE: Effectiveness and safety
   PROBE: Knowledge of Td or Tdap vaccine

3. What do you know about the flu vaccine?

   PROBE: Effectiveness and safety

4. Do you think there should be more recommended vaccines for pregnant women? Why or why not?

   PROBE: Other vaccine preventable diseases
   PROBE: Td or Tdap?
   PROBE: flu?

5. What do you think will create demand for new vaccines in the public and among pregnant women?

   PROBE: Are there any opportunities you have to facilitate this demand?
   PROBE: Where is this demand coming from?

These are all the questions I have for you today. Thank you for taking the time to meet with me today.
