# Supplementary material for: Patient and provider perspectives on how trust influences maternal vaccine acceptance among pregnant women in Kenya
Source: BMC Health Serv Res. 2019 Oct 24;19:747. doi: 10.1186/s12913-019-4537-8 (PMC6813986; doi:10.1186/s12913-019-4537-8)
Supplement: Supplementary file 2 — Additional file 2. Interview guide for pregnant women. [file 12913_2019_4537_MOESM2_ESM.pdf]

Anthropologic Data Collection Narrative Interview Guide

Pregnant Women

Introductory Questions:

1. How old are you?

2. What is your current profession?

3. What is the highest level of education you have completed?

Elicitation of information on beliefs, attitude and immunization behavior in the context of values and motivational factors

1. What are the most important factors you consider when you are making health decisions?

2. Who do you trust regarding information relating to your health?

   PROBE: Healthcare provider, Family, Peers, Media

3. Before this visit, have you seen a healthcare provider for your own health in the last year?

4. What do you know about vaccines?

5. Have you ever received a vaccine before?

   If yes:

   a. Which one/s?

   b. Do you know what the vaccine prevents again?

   c. Why did you receive the particular vaccine/s?

      PROBE: doctor/family told you to

6. Where do you receive your vaccination information from?

7. Are there any particular advertisements or informational items that you particularly liked related to vaccinations?

   PROBE: Where did they see it?
   PROBE: Who was it targeted for?

8. Have you ever refused vaccine for yourself before? Why? Why not?

9. Do you believe that vaccines are safe?

   PROB: kinds of illnesses shots prevent, side effects, immunity development through shots, are there too many/too few shots, fear that shots might not be as effective in preventing disease etc.

10. Do you know anyone who had a bad reaction to vaccines?

11. Do you think (or know of others who think) vaccines themselves make you sick?

12. Would you rank 3 main reasons for you to decide (or not) to get the vaccines?

    PROB: Factors/concerns that motivate or demotivate her.

Maternal and infant Vaccine Questions:

1. What do you know about vaccinations in pregnancy and vaccines for infants?

2. Where did you receive this information from?

3. Would you rank the top 3 most trusted sources of information relating to vaccinations?

   PROBE:

4. Would you get a vaccine for your infant? Why? Why not?

5. Who would you discuss getting your child vaccinated with?

   PROBE: Family, Healthcare Provider, Peers,

6. Do you believe that children should receive vaccines? Why? Why not?

7. Do you believe some mothers should have the liberty to choose NOT to vaccinate themselves and their children? Why? Why not?

8. How would you feel if your child caught a severe illness from another child who was unvaccinated?

Closing Questions:

1. Do you have anything else to say regarding vaccinations?

2. Do you have any questions or comments?

These are all the questions I have for you today. Thank you for taking the time to meet with me today.
